# Mitochondria in skin health, aging, and disease

**DOI:** 10.1038/s41419-020-2649-z

**Published:** 2020-06-09

**Authors:** Annapoorna Sreedhar, Leopoldo Aguilera-Aguirre, Keshav K. Singh

**Affiliations:** 1 Yuva Biosciences, 1500 1st Avenue N, Birmingham, AL 35203 USA; 2 Department of Genetics, University of Alabama at Birmingham, Birmingham, AL 35294 USA; 3 Integartive Center For Aging Research and O’Neal Comprehensive Cancer Center, University of Alabama at Birmingham, Birmingham, AL 35294 USA

**Keywords:** Skin cancer, Metabolic disorders

## Abstract

The skin is a high turnover organ, and its constant renewal depends on the rapid proliferation of its progenitor cells. The energy requirement for these metabolically active cells is met by mitochondrial respiration, an ATP generating process driven by a series of protein complexes collectively known as the electron transport chain (ETC) that is located on the inner membrane of the mitochondria. However, reactive oxygen species (ROS) like superoxide, singlet oxygen, peroxides are inevitably produced during respiration and disrupt macromolecular and cellular structures if not quenched by the antioxidant system. The oxidative damage caused by mitochondrial ROS production has been established as the molecular basis of multiple pathophysiological conditions, including aging and cancer. Not surprisingly, the mitochondria are the primary organelle affected during chronological and UV-induced skin aging, the phenotypic manifestations of which are the direct consequence of mitochondrial dysfunction. Also, deletions and other aberrations in the mitochondrial DNA (mtDNA) are frequent in photo-aged skin and skin cancer lesions. Recent studies have revealed a more innate role of the mitochondria in maintaining skin homeostasis and pigmentation, which are affected when the essential mitochondrial functions are impaired. Some common and rare skin disorders have a mitochondrial involvement and include dermal manifestations of primary mitochondrial diseases as well as congenital skin diseases caused by damaged mitochondria. With studies increasingly supporting the close association between mitochondria and skin health, its therapeutic targeting in the skin—either via an ATP production boost or free radical scavenging—has gained attention from clinicians and aestheticians alike. Numerous bioactive compounds have been identified that improve mitochondrial functions and have proved effective against aged and diseased skin. In this review, we discuss the essential role of mitochondria in regulating normal and abnormal skin physiology and the possibility of targeting this organelle in various skin disorders.

**Facts**

- Skin is the largest organ with high turnover rate in the human body.
- Mitochondria play a vital role in the skin.
- Mitochondrial dysfunction induces skin aging.
- Skin disorders manifest mitochondrial dysfunction.
- Targeting mitochondria may help rejuvenate skin.

**Open Questions**

- How do mitochondria regulate skin aging?
- Is mitochondrial dysfunction a primary or a secondary cause of skin aging?
- Does targeting mitochondria prevent or slow down skin aging?

## Introduction

The skin is the outer sheath of the body, which interfaces with the external environment. It is a complex structure made of several tissue layers, each with a distinct cellular composition. It has crucial functions like sensation, heat insulation and prevention of water loss, and acts as a physical barrier to pathogens. The upper layer of the skin is the epidermis, a stratified structure interspersed with hair follicles. The outer epidermal surface is made of cornified keratinocytes or corneocytes that form a dense cytoskeletal network of keratin filaments. At the same time, the basal layer harbors the epidermal stem and progenitor cells, which continuously regenerate the epidermis. The dermis lies underneath the epidermis and consists of dermal fibroblasts that produce collagen and elastin that form the extracellular matrix (ECM), as well as the melanocytes that produce the photo-protective pigment melanin^1^. With aging, both the epidermis and dermis undergo thinning and lose their regenerative capacity, which manifests as wrinkling, dryness, and mottling. Also, chronic exposure to environmental elements like ultraviolet (UV) radiations cause deleterious effects in the skin cells^2^. UVA and UVB radiation are not only associated with premature skin photo-aging but also various inflammatory reactions and skin cancer^3^.

**Fig. 1:**
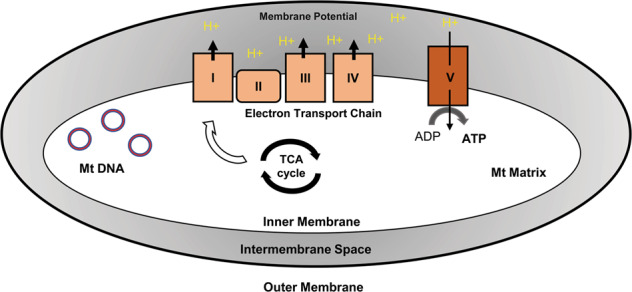
Mitochondria in skin health. Mitochondrion is a double-membrane organelle found in the cytoplasm of almost all eukaryotic cells. It is the site of cellular respiration and most ATP production. Mitochondrial respiration utilizes a series of protein complexes: complex I (NADH dehydrogenase), complex II (succinate dehydrogenase), complex III (cytochrome c reductase) and complex IV (cytochrome c oxidase) located in the inner mitochondrial membrane. These complexes sequentially translocate electrons (e^−^) to create a proton gradient (H^+^). Complex V (ATP synthase) then makes use of this gradient to phosphorylate ADP to ATP.

The skin is a high turnover organ, with a continuously regenerating epidermis. The epidermal progenitors are thus highly proliferative and metabolically active and rely on adenosine triphosphate (ATP) for their energy requirements. ATP is mainly produced through oxidative phosphorylation (OXPHOS) in the mitochondrion, which is the bioenergetics center of the eukaryotic cell^4^. This double membrane-bound organelle is involved in essential functions like energy production, fatty acid oxidation (FAO), heme and steroid biosynthesis, apoptosis, and calcium signaling. Mitochondrial respiration utilizes a series of protein complexes located at the inner membrane, collectively known as the electron transport chain (ETC). These complexes sequentially transport electrons and translocate protons to the intermembrane space creating a proton gradient, which is utilized to generate ATP^5^ (Fig. 1). The natural by-products of OXPHOS include reactive oxygen species (ROS) such as superoxide anion, singlet oxygen and peroxides, which can disrupt macromolecular and cellular structures. Although ROS production can be triggered by external stimuli such as ionizing radiations and carcinogens, the generation of endogenous ROS is highest in the mitochondria^6^. The oxidative damage caused by mitochondrial ROS production is an essential molecular basis of various pathophysiological conditions, including aging and cancer^7^.

**Fig. 2:**
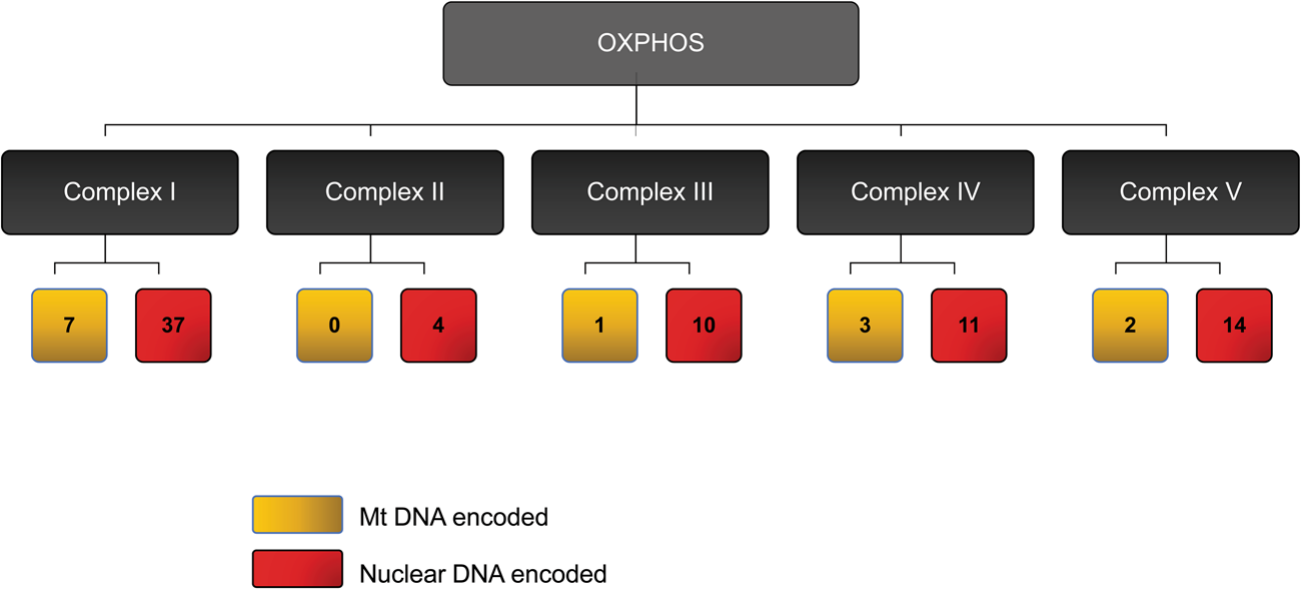
Mitochondrial OXPHOS subunit composition. Schematic representation of mammalian respiratory chain complex. The ETC consists of five complexes, with subunits encoded by both mtDNA (yellow) and nuclear DNA (red). The human mtDNA genome encodes only 13 OXPHOS proteins, while the nuclear DNA encodes the remaining 80% of the OXPHOS proteins.

Mitochondria are unique organelles that possess a maternally inherited autonomous genome. The mitochondrial DNA (mtDNA) encodes for 13 OXPHOS proteins, in addition to 22 tRNAs and two rRNAs^8^ (Fig. 2). Due to their physical proximity to the ETC and therefore, ROS, mtDNA is highly susceptible to mutations, which invariably disrupt OXPHOS, thus triggering a vicious cycle of increased ROS production and mtDNA damage^9^. Somatic mutations in mtDNA are often observed in various diseases, including cancer. Besides, mtDNA exists as multiple copies, and studies have associated abnormal copy numbers with different pathophysiological states^10^. Some mtDNA mutations or single nucleotide polymorphisms (SNPs) can persist in a population, giving rise to haplogroups with distinct geographical distribution; at present, around 27 major mitochondrial haplogroups are known, and many have been correlated with a higher risk of cancers and other diseases, including those of the skin^11^.

**Fig. 3:**
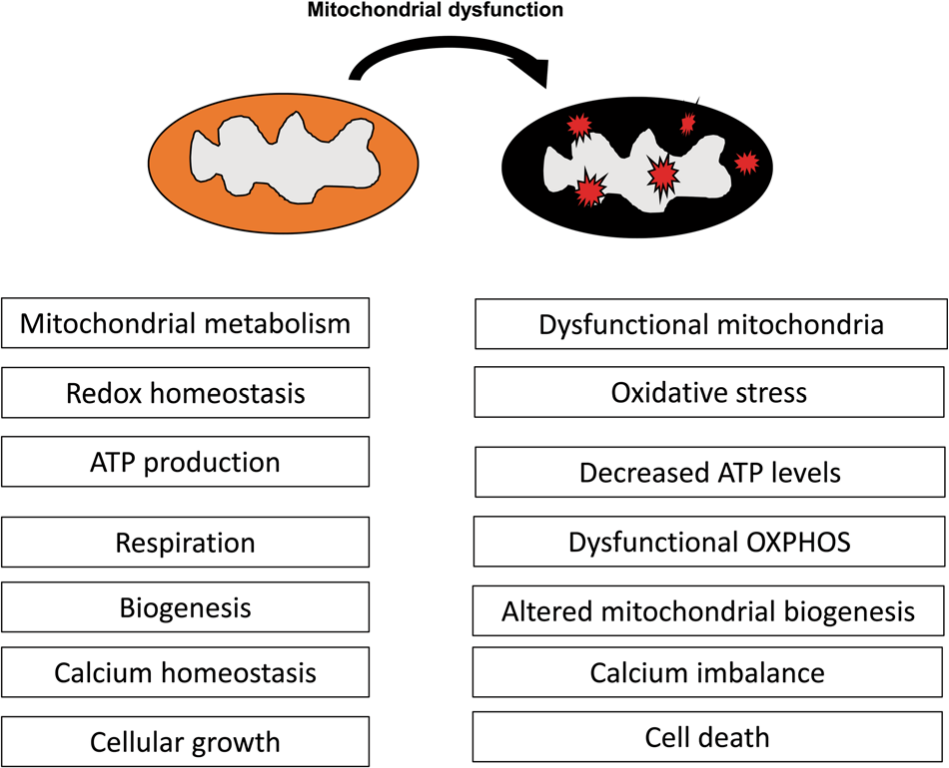
Consequences of mitochondrial dysfunction. Mitochondrial dysfunction is accompanied by enhanced ROS production, reduced antioxidant defense mechanism, altered cellular respiration, altered metabolism, and enhanced cell death.

Recent studies have established an essential role of the mitochondria in skin homeostasis, as well as disorders such as cancer, inflammatory conditions, and rare inherited diseases. The mechanistic basis of mitochondrial dysfunction in the skin and other organs, is primarily ROS production and oxidative stress, although other mitochondrial pathways have also been linked with certain skin disorders (Fig. 3). In this review, the essential role of mitochondria in regulating normal skin physiology, as well as in healthy/premature skin aging and skin cancers, have been discussed. Also, the therapeutic targeting of mitochondria in various skin disorders has been considered.

## Role of mitochondria in skin homeostasis

### Mitochondria-dependent epidermal differentiation

The epidermis is a self-renewing layer that depends on the subsequent differentiation and migration of the proliferative basal keratinocytes to the cornified keratinocytes in the outermost squamous layer^12^. Studies increasingly show that mitochondrial ROS generation plays a regulatory role in the differentiation of various cell lineages via several downstream transcriptional factors like C/EBP, Notch, and β-catenin, and increased ROS levels promote differentiation of murine embryonic stem cells (ESCs), induced pluripotent stem cells (iPSCs), as well as tissue-specific multipotent epithelial stem cells^13^. Hamanaka et al.^14^ found that conditional mitochondrial transcription factor A (TFAM)-knockout mice had a high neonatal mortality rate due to impaired epidermal barrier function and lacked normal fur. Also, primary keratinocytes isolated from these mice failed to differentiate in vitro. Since TFAM drives mtDNA replication and cells lacking TFAM have defective OXPHOS and low ROS levels, it is reasonable to surmise that mitochondrial respiration and ROS production are essential for keratinocyte differentiation. Indeed, antioxidant treatment of wild-type keratinocytes blocked their differentiation, while exogenous administration of H_2_O_2_ restored the same in TFAM^−/−^ keratinocytes. Further mechanistic analysis revealed that mROS promoted epidermal differentiation by activating the Notch and β-catenin signaling cascades. Besides, low levels of H_2_O_2_ generated in the skin fibroblasts exposed to physiological doses of UVB rays activated the keratinocyte growth factor receptor and promoted keratinization.

Mitochondrial respiration also requires Ca^2+^ uptake, and a Ca^2+^ gradient has been observed in the epidermis in vivo^15^. Keratinocyte differentiation in vitro is enhanced by increasing Ca^2+^ levels, whereas the opposite effects are seen upon inhibiting mitochondrial calcium uptake^16^. In a recent study, Bhadhuri et al.^17^ used the gene network-based bioinformatics approach to screen for hub proteins involved in epidermal differentiation and identified the mitochondrial proteins MPZL3 and FDXR. Functional analyses further showed that the proteins co-localized proximally in the keratinocyte mitochondria and promoted their differentiation by inducing ROS generation. Interestingly, depletion of the myelin protein zero-like 3 (MPZL3) is associated with dermatitis and alopecia in mice, and the ETC enzyme ferredoxin reductase (FDXR) sensitizes various human epithelial cell lines to ROS-induced apoptosis. Allombert-Blasie et al.^18^ showed that the terminal differentiation of keratinocytes was accompanied by the activation of the mitochondrial caspase-dependent apoptotic pathway

### The melatonin-mitochondria axis in the skin

Melatonin (N1-acetyl-5-methoxytryptamine) is a neuro-hormone secreted by the pineal gland that regulates the circadian rhythm in mammals. It is also synthesized in other organs like the retina, cochlea, lungs, liver, kidney, immune system, and the skin, and its levels are tightly regulated by both hepatic and epidermal metabolism^19^. It has a pivotal photo-protective role in the cutaneous tissues, in addition to regulating wound healing, pigmentation, inflammation, and immune responses. Melatonin and its metabolites directly act on skin cells expressing the G protein-coupled melatonin type (MT) 1 and 2 receptors following UV or X-ray exposure, and attenuate macromolecule damage by scavenging the radiation-induced free radicals^20^. Pharmacological doses of melatonin protect keratinocytes and melanocytes from UVB-induced damage in vitro^21^. Full-thickness human skin histo-cultured ex vivo showed significantly lower levels of the DNA damage marker 8-hydroxy-2′-deoxyguanosine (8-OHdG) following UV exposure when pre-treated with melatonin^22^. Apart from direct scavenging of ROS like H_2_O_2_, singlet oxygen, and NO^−^, melatonin and its derivatives also protect the skin cells from radiation damage by increasing the activity of antioxidant enzymes^22,23^. Interestingly, high levels of endogenous melatonin have been detected in human and rodent skin cells, including melanocytes, keratinocytes, and epithelial cells, in addition to the enzymes involved in melatonin synthesis and metabolism^19^.

The potent radio-protective and antioxidant role of melatonin in the skin raises the possibility of mitochondrial involvement. Indeed, studies have increasingly pointed to a “symbiotic” relationship between melatonin and epidermal mitochondria, with the latter serving as the site of cutaneous melatonin biosynthesis and metabolism. In contrast, melatonin has regulatory effects on the mitochondrial ETC and antioxidant machinery. Melatonin is metabolized in the mitochondria via the cytochrome c and H2O2-dependent kynurenic pathway, and the cytochrome P450-dependent indolic pathway^19,24^. The primary metabolites of melatonin include N1-acetyl-N2-formyl-5-methoxykynuramine (AFMK), 6-hydroxylmelatonin, and N-acetylserotonin, which accumulate in the epidermis following UVB exposure in a dose-dependent manner^19^. A melatonin biosynthetic machinery may also be present in the mitochondria since oocyte mitochondria can synthesize melatonin from serotonin in a cell-free system^25^. It remains to be elucidated whether cutaneous mitochondria also have a similar function. Melatonin can directly increase the electron flux through the respiratory chain and enhance ATP production by donating electrons. One study showed that 6-hydroxymelatonin promoted electron transfer from complex III to complex IV (cytochrome c oxidase), which may help restore the age-dependent decline in mitochondrial bioenergetics. Besides, melatonin mediates the reduction of cytochrome c and augments H_2_O_2_ scavenging^26^.

The intracellular Ca^2+^ homeostasis-regulating protein complex calmodulin also binds to melatonin, indicating the role of the latter in mitochondrial functions like autophagy and apoptosis as well^27^. One study showed that topical application of melatonin improved the barrier function of the epidermis by increasing keratinocyte proliferation^28^. It can also accelerate wound healing and augment the effects of antimicrobials^29^. Given that ROS production also plays a role in tissue repair and regeneration^30^, the melatonin-mitochondrial axis may also be the driver of epidermal wound healing.

## Mitochondrial role in skin aging

In addition to the inevitable time-induced changes, the skin is also highly susceptible to photo-aging due to chronic exposure to solar UVA and UVB radiations^2^. Aging affects the protective role of the skin against physicochemical and biological attacks, as well as its thermoregulatory, sensory, immunological, and hormonal functions. The structural and functional manifestations of cutaneous aging are premature and more severe in the photo-aged compared to the chronologically aged skin. Furthermore, since photoaging is a cumulative process, it is more pronounced among older individuals who have been regularly exposed to the sun for long periods. Both intrinsic and environmental factors affect the epidermal and dermal layers of the skin. Histologically, chronological aging is characterized by significant epidermal thinning, which manifests as dryness and wrinkles^1^.

**Fig. 4:**
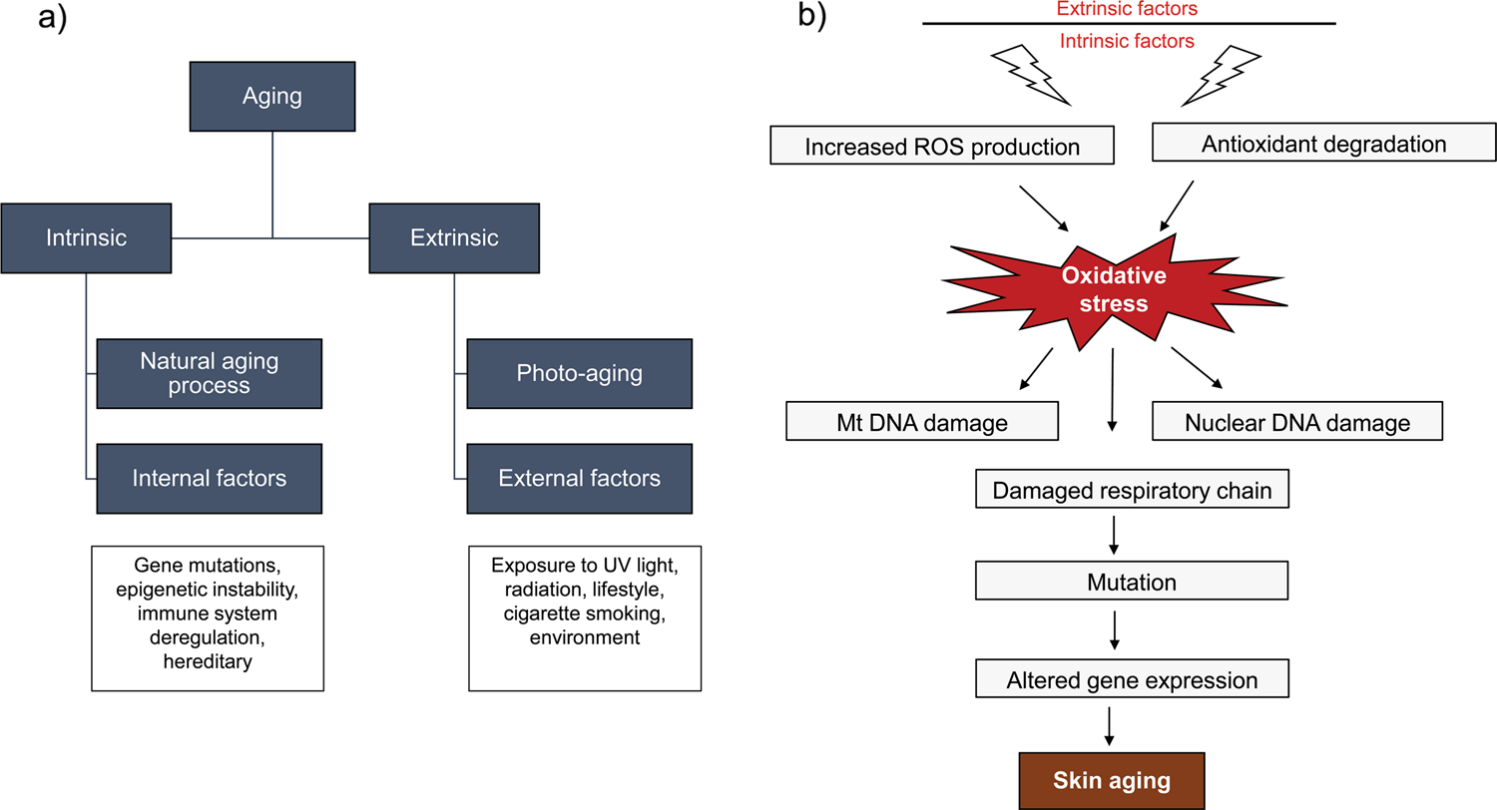
Mitochondria in skin aging. **a** Skin aging categories: Intrinsic (internal) aging and extrinsic (external) aging. **b** Skin aging is characterized by loss of membrane potential, elevated levels of ROS, DNA damage leading to nuclear and mitochondrial gene mutations, respiratory chain defect due to enzyme alterations, altered cellular regulation, and disease progression.

In contrast, photo-aged skin has a thick, leathery appearance with deeper wrinkles and uneven pigmentation^31^. Several studies have directly or indirectly linked mitochondrial dysfunction to both chronological and photo-aging of the skin^32^. At the molecular levels, aged skin is characterized by damaged mitochondria, mtDNA deletions, high ROS levels, and oxidative stress in both the dermal and epidermal layers (Fig. 4).

### Chronological skin aging

Mutations and deletions in mtDNA are known to accumulate in post-replicative tissues with aging, which is accompanied by a steady decline in mitochondrial function^33^, increased ROS production, and loss of mitochondrial membrane potential (MMP), followed by increased mitophagy, and apoptosis. A 4977 base pairs extended region of mtDNA, including the genes encoding complexes I, IV, and V of the respiratory chain, is frequently deleted in the aged human skin^34^. This “common deletion” is more evident in the skin of individuals older than 70 years and increases with sun exposure^34^. Kaneko et al.^35^ compared the neck skin samples of German and Japanese women and found a general increase in the frequency of the common deletion with age, and in sun-exposed versus photo-protected skin. Besides, a strong positive correlation was observed between the density of common deletion and skin wrinkles among the Japanese women, while the higher frequency of common deletion correlated with fewer pigmented lesions among the German women. The latter also showed a slightly higher amount of the common deletion compared to Japanese women, which may be due to intrinsic genetic variations, or different cultural attitudes towards sun exposure. In a recent study, Singh et al.^36^ established an inducible mtDNA-depletion mouse model with a dominant-negative mutation in the mtDNA polymerase *POLG1*. The mice had ubiquitous mtDNA depletion and correspondingly reduced OXPHOS. The phenotypic manifestations included extensive skin wrinkling and fur loss, which in turn were associated with epidermal hyperplasia and hyperkeratosis. The transgenic mice had increased levels of matrix metalloproteinases that are often increased during skin aging. Interestingly, turning off the mutant *POLG1* expression restored the cutaneous pathologies to the wild-type level. This study is the first to prove that mtDNA depletion is the underlying cause of skin aging and that restoring mitochondrial functions can restore skin youthfulness.

The age-dependent accumulation of ROS in the keratinocytes, and the concomitant loss of MMP, results in a metabolic shift from OXPHOS to the anaerobic glycolysis. Prahl et al.^37^ isolated keratinocytes from skin biopsies of old and young donors and found a distinctly glycolytic phenotype of the older keratinocytes, and addition of the ETC component coenzyme (Co) Q10 restored mitochondrial metabolism in the aged cells. Consistent with this, an age-related decline in complex II (succinate oxidoreductase) activity has also been observed in aged human skin fibroblasts^38^. Damaged mitochondria are cleared away by a highly conserved pathway called mitophagy, or the selective autophagy of mitochondria^39^. Mitophagy levels increase considerably after cellular stress or damage, and homeostasis between mitochondrial biogenesis and mitophagy is vital for a healthy mitochondria pool. Aymard et al.^40^ demonstrated a critical role of autophagy and mitophagy in keratinocyte differentiation, which also raises the possibility of mitochondrial fragmentation in aged keratinocytes owing to the increase in ROS levels with aging and elevated mitochondrial fission in response to oxidative stress. Recently, Mellem et al.^41^ studied the mitochondrial network in young and old human skin for the first time in vivo and found significantly fewer mitochondrial clusters in the keratinocytes. A highly connected physical network of mitochondria in the epidermal cells of the younger compared to the older skin. The latter had a significantly fragmented mitochondrial network, indicating poor recycling and excessive mitophagy. The similarities in mitochondrial dynamics in normal differentiation and aging could either be due to common pathways that are dysregulated during aging or simply due to the aging-related reduced epidermal turnover^42^.

Coenzyme Q (CoQ10) is a lipophilic isoprenylated quinone that acts as an electron shuttle between complex I/II and complex III of the ETC, and as a ROS scavenger that protects against membrane lipid oxidation^43^. Both the antioxidant and bioenergetic roles of CoQ10 are closely associated with skin aging and other disorders. CoQ10 levels are 10-fold higher in the epidermis compared to the dermis and decrease significantly with age. Reduced CoQ10 content in aged dermal fibroblasts is associated with lower activity of the complexes I/III and II/III, membrane depolarization, and generation of superoxide anions^44^. Furthermore, several studies have shown that topical application of CoQ10 on photo-aged skin ameliorates the phenotypic signs of aging and restores mitochondrial function^45^. The age-related glycolytic shift reported by Prahl et al.^37^ in the human keratinocytes was also associated with impaired CoQ10 function and was reversed by its exogenous application. CoQ10 is synthesized de novo by the mevalonate pathway and can be inhibited by 3-hydroxy-3-methyl-glutaryl-coenzyme A (HMG-CoA) reductase inhibitors like statins. Marcheggiani et al.^46^ recently showed that statin mediated CoQ10 inhibition in human dermal fibroblasts triggered oxidative stress and mitochondrial dysfunction, and lead to premature “aging” of the cells in vitro.

### Photo-aging

Chronic UV exposure induces nuclear and mitochondrial DNA damage and oxidative stress in the skin cells, which can progress to photo-aging and or skin cancer. UVB mainly acts on the epidermal keratinocytes and melanocytes, while UVA can penetrate more deeply into the dermis^47^. A cardinal marker of photo-aging, which is relatively less obvious in chronological aging is large-scale mtDNA deletion. Berneberg et al.^48^ reported a 10-fold higher rate of a ∼5000 bp deletion in the mtDNA of photo-aged compared to sun-protected skin cells. In a later study, the same group^49^ found that repeated exposure of the normally unexposed buttock skin to UVA increased the frequency of mtDNA deletions in the dermal cells by 40% compared to the non-irradiated skin.

Interestingly, these UV-induced deletions can persist over many years and can increase by 30–40 folds even in the absence of further irradiation^47,48^. UVA/B exposure, however, is not homogenous across the entire epidermis, since some parts of the body are more frequently exposed to the sun than others. Krishnan et al.^50^ observed a significantly higher frequency of a 3895 bp mtDNA deletion block in habitually exposed areas of the skin compared to the less exposed regions. In a recent study by Powers et al.^51^ an unusually high rate of the common mtDNA deletion (4977 bp) and a 3895-bp deletion was observed in the sun-exposed skin biopsies of elderly individuals that were diagnosed negative for any skin cancer.

Repeated exposure to sub-lethal doses of UVA resulted in mtDNA deletions in primary human keratinocytes^52^ and dermal fibroblasts, which was accompanied by decreased mitochondrial function, increased activity of collagen-degrading enzymes^53^ and oxidative stress, which further exacerbated the mitochondrial damage^54^. Schroeder et al.^55^ generated mtDNA-depleted human skin fibroblast lines. They found that in addition to aberrantly high oxidative stress, these cells showed the typical molecular features of photo-aged skin, such as increased levels of collagen degrading metalloproteases (MMPs) and downregulation of the genes involved in collagen biosynthesis^45^, which translates to the extensive wrinkling of photo-aged skin^31^. This is consistent with the hypothesis of altered collagen metabolism in photo-aging, first proposed by Fisher et al.^46^. Furthermore, skin fibroblasts isolated from patients with Kearns–Sayre syndrome (KSS), a rare inherited disorder characterized by extensive mtDNA deletions, showed higher ROS levels and increased activity of photo-aging-related genes^56^. Oyewole et al.^57^ compared the protective effects of mitochondria-specific antioxidants and cellular antioxidants (resveratrol and curcumin) against UVA-induced mtDNA damage in human dermal fibroblasts. They detected significantly higher photo-protection by the former. These findings collectively indicate that UV-induced photo-aging is directly linked to mtDNA depletion and that the phenotypic features of photo-aged skin are the result of mitochondrial dysfunction and ECM degradation.

In addition to the UV radiation, infrared (IR) rays have also been associated with photo-aging of human skin. Schieke et al.^58^ showed that exposure to IR dysregulated the expression of metalloproteases in primary human fibroblasts. Similar changes were observed *in situ* in human skin exposed to IR^54^, while chronic exposure to IR also resulted in the typical clinical signs of photo-aging in animal models^59^. Schauen et al.^60^ detected a significant increase in the intra-mitochondrial levels of ROS in primary human skin fibroblasts exposed to IR, indicating a direct role of the mitochondria in IR-induced photo-aging. Consistent with this, treatment of IR-exposed human fibroblasts with either an antioxidant or an ETC inhibitor reversed the signs of photo-aging^61^. The underlying mechanism of IR-induced mitochondrial damage was elucidated by Karu et al.^62^ who showed that the IR rays are absorbed by the copper atoms of ETC complex IV, leading to disturbed electron flow and ‘leakage,’ and increased ROS production.

Mitochondrial perturbations, such as mtDNA mutations/deletions, loss of MMP, and the uncoupling of the OXPHOS machinery induces mito-nuclear retrograde signaling. The effect of retrograde signaling is a global alteration in nuclear gene expression, which leads to metabolic shift, apoptosis, and mitophagy. In recent years, retrograde mitochondrial signaling has been directly linked with various pathological states. In the context of photo-aging, retrograde mitochondrial signaling alters the expression of nuclear genes involved in collagen biosynthesis and degradation, inflammation, and neovascularization, all of which contribute to the appearance of the photo-aged skin^61^. The presence of oxidized proteins in the upper dermal layers of photo-damaged skin lends support to this hypothesis^63^.

While chronological aging is associated with a 10–20% decrease in melanin production per decade, chronic UV exposure increases the amount of this photo-protective pigment, resulting in the typical “mottled” appearance of photo-aged skin^64^. Melanocytes are highly susceptible to oxidative damage since melanogenesis is pro-oxidative and have higher basal levels of ROS compared to other cell types. Melanocytes from UV-exposed skin show an increase in ROS and melanin levels compared to the cells isolated from normal skin. While endogenous melanin is known to protect melanocytes from photo-oxidative damage by absorbing the UV rays, chronic UV exposure can lead to melanin oxidation, which in turn increases ROS levels and further aggravates the oxidative damage. There is evidence indicating that the melatonin-regulated cutaneous circadian clock also controls melanogenesis in the human epidermis and cultured fibroblasts^65^.

## Mitochondrial role in skin cancer

Skin cancer is the most common malignancy affecting humans and accounts for 40% of all cancer cases worldwide. It is classified into the non-melanoma skin cancers (NMSCs) that include basal-cell carcinoma (BCC) and squamous cell carcinoma (SCC), and the melanocyte-derived melanomas. Although the latter comprise only 5% of all skin cancer cases, they are associated with a significant mortality rate of ∼25% as well as metastasis risk^66^. Skin cancer risk is linked to UV radiation, and the incidence of all skin cancer types have significantly increased in the last 2–3 decades due to increased exposure to UV rays, both due to the thinning ozone layer and frequent use of tanning beds. The risk of developing skin cancer, especially malignant melanoma, is also higher in the Caucasian population on account of their lower cutaneous melanin content^67^. Mitochondria have been implicated in almost all human cancers, primarily on account of their role in ROS generation and apoptosis. Congenital and somatic mutations in mtDNA have been detected in most tumors, including those affecting the skin^68–70^, and tumorigenic stimuli such as radiation and carcinogens are known to damage the mtDNA directly^71–75^. Furthermore, mounting evidence suggests that mitochondria are involved in the interplay between oxidative stress, hypoxia^76–81^, and metabolic reprogramming of tumor cells^82–86^.

## Mitochondrial involvement in other skin conditions

**Fig. 5:**
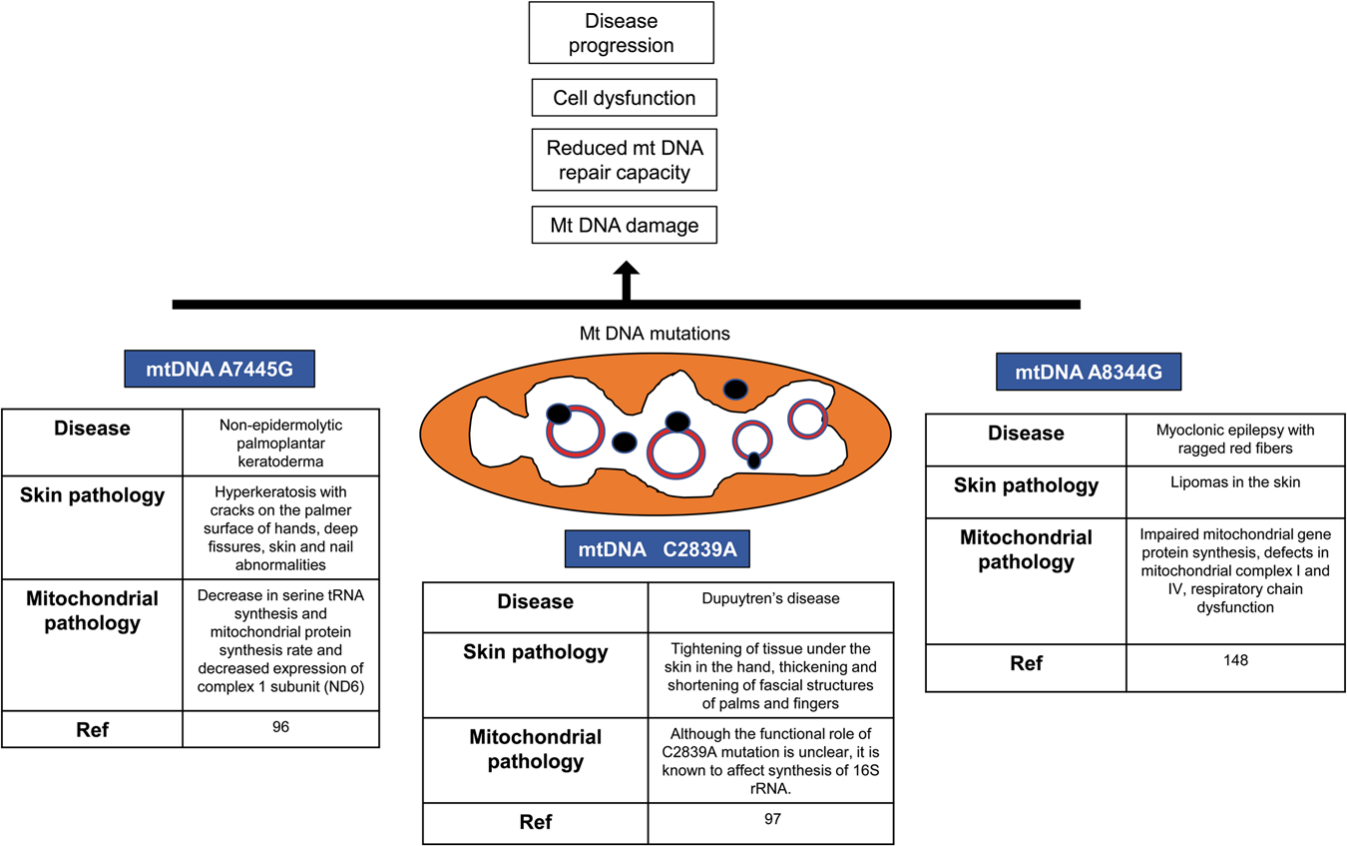
Skin disorders caused by mutations in mtDNA encoding mitochondrial proteins. Skin disorders are a heterogeneous group of diseases caused by mutations in mitochondrial (this figure) and nuclear DNA (Table 1).

In addition to skin aging and cancer, mitochondrial dysfunction has also been associated with various common and rare skin disorders that can be broadly classified into three types: dermal manifestations of primary mitochondriopathies, skin diseases due to mitochondrial dysfunction, and cutaneous manifestations of genetic diseases that affect mitochondria. Clinically, these disorders can be present as hair abnormalities, inflammation, rashes, hypo- and hyperpigmentation, and acrocyanosis (i.e., blueness of extremities). The underlying molecular mechanisms have been identified for most, and mainly involve dysfunctional OXPHOS and high ROS generation, mutations in mtDNA, an imbalance between mitochondrial biogenesis and mitophagy, and aberrant expression levels of nuclear-encoded mitochondrial proteins. In addition, dysregulation in other mitochondrial metabolic pathways and structural proteins have also been implicated. The entire gamut of skin diseases with direct or indirect mitochondrial involvement has been summarized in a recent review by Feichtlinger et al.^87^ (Fig. 5 and Table 1).

**Table 1:** Skin disorders caused by mutations in nuclear DNA affecting mitochondrial functions.

Gene	Skin pathology	Mitochondrial pathology	Ref.
Ataxia-Telangiectasia Mutated (ATM)	Telangiectasia, progeric and sclerodermatous skin changes	Mitochondrial abnormalities, elevated ROS, increased aberrant mitochondria, respiratory enzyme deficiency, defective mitochondrial respiration, decreased mitophagy.	^105,117–119^
Biotinidase (BTD)	Hypotonia, skin rashes, hair loss	Defective biotin metabolism, carboxylase and biotinidase deficiency, ATP depletion, defective TCA cycle.	^99,120–122^
Mitochondrial chaperone (BCS1L)	Hypertrichosis, abnormal hair growth, brittle hair	Mitochondrial complex III deficiency, defective mitochondrial respiration, changes in mitochondrial morphology, mitochondrial encephalopathy, multiple organ failure	^123–125^
Cytochrome c oxidase 7B (COX7B)	Microphthalmia with linear skin lesions, aplastic skin, hyperpigmented areas on the skin, skin defects, linear areas of erythematous skin hypoplasia	OXPHOS defects, mitochondrial respiration deficiency, CNS defects, developmental deficiencies	^126^
Coproporphyrinogen oxidase (CPOX)	Photosensitivity, skin lesions, jaundice	Decreased activity of CPOX, mitochondrial abnormalities, acute ataxia and iron-sulfur assembly defects.	^100,127–129^
Comparative gene identification-58 (CGI-58)	Congenital ichthyosiform erythroderma, abnormal scaling of the skin with underlying redness.	Abnormal accumulation of intracellular lipid droplets in nonadipose tissues, dysregulated lipid homeostasis, myopathy, impaired fatty acid oxidation, increased mitochondrial size, reduced mtDNA content	^130,131^
DNA excision repair protein ERCC6 (ERCC6)	Freckled skin, scarring, pigmentation, atrophy, anhidrosis, decreased subcutaneous adipose tissue	Increased ROS, accumulation of damaged mitochondria, defective mitophagy, incompetent DNA repair system, altered cellular homeostasis.	^132,133^
Fanconi anemia complementation group A (FANCA)	Pigmentary changes, short stature, hyper pigmented skin, absence of left-hand thumb	Reduced electron transfer between respiratory complex I-III, reduced ATP/AMP ratio, deficiency of ROS detoxification enzymes.	^134,135^
Ferrochelatase (FECH)	Light-sensitive dermatitis, photosensitivity leading to skin thickening and areas of hyperkeratosis, skin itching and burning, erythema, edema, mild scarring	Reduced activity of FECH, mitochondrial abnormalities, iron-sulfur assembly defects	^127–129^
Human HRas (HRAS) or transforming protein p21	Loose redundant skin, coarse facial features, dark skin pigmentation, papillomas, abnormal thickening and darkening of skin, deep palmar creases	Inactive HRAS protein, uncontrolled cell division, altered metabolism, mitochondrial dysfunction, defective OXPHOS	^136,137^
Halocytochrome c-type heme lyase synthase (HCCS)	Skin defects limited to the face and neck, aplastic skin, appearance of skin lesions, hyperpigmented areas of the skin	HCCS deficiency, enhanced cell death, reduced OXPHOS efficiency	^138^
Isocitrate dehydrogenase (IDH1; IDH2)	Enchondromata, small red scratches or bumps on the skin, hemangiomata	Inactive IDH enzyme, decreased α‐ketoglutarate levels and increased accumulation of oncometabolite 2‐hydroxyglutarate, increased oxidative stress, dysfunctional TCA cycle	^95^
Protoporphyrinogen oxidase (PPOX)	Skin photosensitivity, skin hyperpigmentation and hypertrichosis	Reduced activity of enzyme PPOX, mitochondrial abnormalities, iron-sulfur assembly defects	^127–129^
Lamin A/C (LMNA)	Early loss of hair, patchy skin coloring, loss of fatty tissue under the skin, particularly affecting the limbs	Swollen mitochondria with loss of cristae, reduced mitochondrial function, decreased ATP generation, altered metabolism,	^139^
Menkes’ protein (MNK)	Hypopigmentation, loose and sagging skin, coarse hair, loose joints	Reduced activity of MNK, reduced heme synthesis, diminished complex IV activity, dysfunctional OXPHOS	^101^
Plectin 1 (PLEC1)	Skin blistering at birth or shortly thereafter, progressive muscle weakness, deformities of the nails, palmoplantar hyperkeratosis, and alopecia	Aberrant plectin expression, reduced skeletal muscle function and network, increased skin fragility, muscular weakness, muscular dystrophy, myopathy, structural and functional alterations of mitochondria, reduced complex II, IV and V activity, diminished OXPHOS	^102,140,141^
Pyrroline-5-carboxylate reductase 1 (PYCR1)	Loose skin, loss of bone density	Altered mitochondrial morphology, increased oxidative stress, increased cell death	^142,143^
ATP-dependent DNA helicase Q4 (RECQL4)	Skin rashes, hair loss, skin lesions, poikiloderma, threadlike red lines or patterns on the skin, atrophy, sun hypersensitivity, ulcers	Altered mitochondrial integrity and mitochondrial bioenergetics, loss of mitochondrial reserve capacity, accumulation of damaged mtDNA	^144^
RNA component of mitochondrial RNA processing endoribonuclease (RMRP)	Hypoplastic hair	Defective mitochondrial RNA-processing endoribonuclease	^145^
SAM domain and HD domain-containing protein 1 (SAMHD1)	Skin rash, bluish or purple coloring of the hands and feet, photosensitivity, purpura, petechia, jaundice	Replication defects, defective mitochondria DNA synthesis, altered dNTP pool, complex I deficiency	^146^
Pemphigus vulgaris (PV) caused by MtABs	Skin erosions or blistering formations on the skin	Increased ROS, altered mitochondrial membrane potential, proton leakage, OXPHOS defect	^110^
Sytemic lupus erythematosus (SLE) caused by MtABs	Skin inflammation, red rash, swollen joins, hair loss	Mitochondrial dysfunction in T cells / lymphocytes, elevated ROS, hyperpolarized mitochondria, ATP depletion	^69,108^
BRAF, NRAS, KIT, GNAQ, CDK4, PTK2B, ERBB4, GNA11, MEK, MITF, AKT3, MMP, CXCR4, EPHA3, FAS, PIK3CA, MET, CTNNB1, NEK10, PDGFRA	Melanoma, asymmetry of skin architecture, skin pigmentation, change in skin color, size and diameter, nuclear atypia	Mitochondrial dysfunction, mitochondrial respiratory complex deficiency, elevated ROS levels, dysregulated antioxidant defense system, decreased mitochondrial OXPHOS and increased Warburg effect.	^70,147,148^
PTCH1; PTCH2	Basal cell carcinoma, erythematous patch, papule, nodule or plaque which is often eroded, ulcerated or indurated,	Mitochondrial dysfunction, mitochondrial respiratory complex deficiency, elevated ROS levels, dysregulated antioxidant defense system, decreased mitochondrial OXPHOS, altered metabolism.	^149^

### Skin manifestations of congenital mitochondriopathies

Although congenital mitochondrial diseases are primarily neuro-musculopathies, various organs are dependent on mitochondrial respiration and therefore affected by organelle dysfunction. Several reports have surfaced over the years regarding the cutaneous involvement of mitochondrial syndromes, although the results are heterogeneous and lack consensus. Bodemer et al.^88^ observed 14 children with assorted mitochondria-related disorders (e.g., myopathies, Leigh syndrome, deafness, Wolfram syndrome, hypotonia, Pearson syndrome, etc.), and detected specific skin and hair abnormalities like alopecia, brittle hair, rashes, mottled pigmentation, erythematous lesions, acrocyanosis, hypertrichosis, etc. Abnormal OXPHOS was confirmed using cultured skin fibroblasts from all patients, with abnormal ETC protein expression in 3 cases and mtDNA aberrations in one case. The authors surmised that a mitochondriopathy should be considered if sudden and unexplained cutaneous signs manifest along with an unrelated disorder. Sonam et al.^89^ assessed 25 children with Leigh syndrome, a neuro-metabolic disorder involving both mitochondrial and nuclear mutations, and observed hypertrichosis and hypopigmentation in most patients. Two mtDNA mutations—T3271C and A3243G—in the MELAS syndrome are associated with nail disorders^90^. Hypomelanosis has also been observed in some cases of Kearns–Sayre syndrome, a multi-organ mitochondrial disorder characterized by large mtDNA deletions^91^. Alper’s syndrome is a rare, autosomal recessive neurological disorder caused by mutation(s) in the *POLG1* gene. Campuzano-García et al.^92^ reported a case of a 10-month-old boy with Alpers syndrome in Mexico (2015) that showed hyperpigmented macules with hypochromic centers in the skin folds of the armpits, groin, and foot dorsum. The rashes were bilateral and mostly concentrated over the dorsal regions. AlSaman et al.^93^ reported a case of a hypotonic 12-month old infant in Saudi Arabia with mtDNA depletion syndrome (MDS) who also showed sparse and brittle hair, scaly skin, eczema, and reticular pigmentation.

The Hutchinson–Gilford progeria syndrome (HGPS) is a very rare congenital disease caused by a dominant mutation in the lamin A gene, which encodes for a structural protein that maintains mitochondrial shape. The cutaneous symptoms of HGPS include hair loss (alopecia), extensive skin wrinkling, and loss of subcutaneous fat that further lends an aged appearance. A recent study on a progeroid patient in Bangladesh reported novel maternally inherited mtDNA mutations, especially in the D-loop region^94^, which correlates with reduced OXPHOS and mitochondrial dysfunction seen in dermal fibroblasts isolated from HGPS patients^95^.

### Skin diseases caused by mitochondrial dysfunction

Several skin diseases are associated with mutations in mitochondrial proteins encoded by mitochondrial or nuclear genes or other aberrations in mtDNA that lead to disrupted OXPHOS, excessive ROS generation, mitochondrial fragmentation, which directly results in the cutaneous symptoms. For instance, non-epidermolytic palmoplantar keratoderma (NEPPK) is associated with the A7445G mutation in mtDNA, which affects the ETC complex IV subunit 1, and decreases cellular respiration^96^. Dupuytren’s disease, a maternally inherited condition characterized by abnormal thickening of the skin of the hands, is caused by a mutation in mtDNA 16S rRNA^97^. The molecular basis of several skin diseases is reduced mitochondrial respiration due to mutations in the nuclear genes encoding OXPHOS proteins. For instance, mutations in the complex IV-encoding nuclear gene *COX7B* is responsible for the rare autosomal dominant aplasia cutis congenita (APLCC), which is typified by the congenital absence of the epidermis^98^. Besides, a defective complex III due to mutated BC1 (ubiquinol–cytochrome c reductase) synthesis-like protein is the genetic basis of the Bjornstad syndrome, an autosomal recessive disease characterized by twisted hair shaft and brittle hair^99^. The iron-porphyrin complex heme is essential for cytochrome c function, and the heme synthesis pathway involves both nuclear and mitochondrial proteins. Mutations in genes encoding for heme biosynthesis enzymes often result in photosensitivity and skin inflammatory conditions like dermatitis^100^. The Krebs cycle provides essential metabolites for heme synthesis, and its disruption has been linked with alopecia and pigmented rashes. Epidermolysis bullosa simplex (EBS), a unique skin condition characterized by recurrent epidermal blistering following minor abrasions or bruises, is caused by mutations in the *Plec1* gene that encodes for the outer mitochondrial membrane protein plectin. *Plec1* mutations not only result in abnormally shaped mitochondria but also complex I and complex IV inhibition^101^. Mutations in the mitochondrial pyrroline-5-carboxylate reductase 1 protein result in extensive fragmentation of the mitochondrial network, which is the genetic basis of autosomal recessive cutis laxa type IIB (ARCL2B), a progeroid skin syndrome characterized by wrinkled and sagging skin^102^.

### Skin manifestations of genetic diseases affecting the mitochondria

Mutations in DNA repair genes indirectly lead to mitochondrial dysfunction due to the accumulation of defective mtDNA and form the genetic basis of multiple congenital disorders with skin involvement. For instance, the Rothmund–Thomson syndrome, which presents cutaneous symptoms like sparse eyebrows and eyelashes, is caused by a mutated helicase RecQ protein-like 4 (*RECQL4*). The absence of *RECQL4* is associated with the accumulation of mtDNA damage and a compensatory increase in mtDNA copy number^103^. Mutations in the *RECQL2* ligase are known to increase mitochondrial ROS production and are the cause of Werner syndrome, a rare autosomal recessive adult progeria with considerable skin involvement^104^. The genetic basis of the neurodegenerative disorder ataxia-telangiectasia (AT) is the loss of the AT mutated (*ATM*) gene, which is the master regulator of the DNA damage response. There is significant heterogeneity in its symptoms, and some affected individuals show premature graying of hair, vitiligo, blotchy pigmentation, and inflamed granulomatous lesions. Lack of the *ATM* gene not only causes massive genomic instability but also impairs mtDNA repair, resulting in reduced OXPHOS and increased mitophagy^105^. There is strong evidence linking aging-associated telomere shortening and impaired mitochondrial metabolism. Dyskeratosis congenita (DKC) is a progressive dermatological condition characterized by premature graying and wrinkling, nail dystrophy, and abnormal pigmentation, and caused by mutations in the telomerase RNA component (*TERC*) and telomerase reverse transcriptase (*TERT*) genes^106,107^. Although there is no direct evidence, the significant increase in oxidative stress in cells harboring the above mutations indicate mitochondrial involvement. In addition to impaired mtDNA repair, autoantibodies against mitochondrial proteins (mtABs) also result in mitochondrial dysfunction. High levels of mtABs against multiple mitochondrial proteins have been detected in autoimmune skin disorders like systemic lupus erythematosus (SLE) and pemphigus vulgaris^108,109^. Consistent with this, keratinocytes isolated from pemphigus vulgaris patients show increased ROS levels and impaired OXPHOS^110^.

## Mitochondrial targeting for skin regeneration

Therapeutic targeting of mitochondria in the skin involves either boosting ATP production or scavenging the excess amounts of free radicals. For instance, numerous studies have demonstrated the anti-aging effects of CoQ10 on cultured human dermal fibroblasts^44^. It is a critical ingredient in many anti-aging and regenerative skin creams, and as shown by Knott et al.^111^ topical application of two different CoQ10-containing formulas significantly replenished the levels of this antioxidant in the dermal and epidermal layers of older skin (>60 years). Photo-stressed skin benefitted from CoQ10 application on account of reduction in *in situ* free radical levels. Schniertshauer et al.^112^ further showed that CoQ10 could restore ATP production, prevent mitophagy, and alleviate oxidative stress in aged skin cells. Nicotinamide, also known as Vitamin B3 or niacinamide, is the precursor of NAD+, which is required for mitochondrial ATP production. The deficiency of nicotinamide causes pellagra, which is characterized by photosensitive dermatitis. The topical application of nicotinamide has shown anti‐inflammatory effects against rosacea, acne, autoimmune skin conditions, and hyperpigmentation. It is a popular additive in “high-end” formulations for regenerating aged and sun-damaged skin^113^. Vitamin C or ascorbic acid is a potent antioxidant that is used in various anti-wrinkle creams to increase collagen synthesis, and topical application of 10% Vitamin C reversed UV-induced oxidative damage by scavenging ROS^114^. There is evidence that resveratrol directly improves mitochondrial function in various cell types by increasing organelle biogenesis and attenuating ROS production^115^. In addition to cosmetic problems, the treatment of inflammatory and other serious skin diseases also involves mitochondrial targeting. For instance, the anti-inflammatory glucocorticoids that are used to treat SLE and pemphigus vulgaris increase mitochondrial membrane potential at low doses but reduce mitochondrial function at high doses^116^. Taken together, bioactive compounds targeting the mitochondria have proved effective against age-related as well as UV-induced skin damage, in addition to different skin diseases with mitochondrial involvement.
